# Developing and using a School Menu Healthiness Assessment Tool to analyse school food provision in Wales

**DOI:** 10.1017/S1368980025000047

**Published:** 2025-01-10

**Authors:** Alice Gilmour, Ruth Fairchild

**Affiliations:** School of Sport and Health Sciences, Cardiff Metropolitan University, Cardiff, UK

**Keywords:** Diet quality, School food standards, Nutrition assessment, Instrument

## Abstract

**Objectives:**

To design and develop a new, innovative and valid School Menu Healthiness Assessment Tool that is suitable for the quantitative and qualitative analysis of school food and drink provision. Second, to analyse primary and secondary school menus and price lists pan-Wales to ascertain their healthiness and whether free school meal (FSM) eligible pupils can afford to access healthy, nutritious food across the school day.

**Design:**

Codable items and categories of school food and drink provision were operationalised before the tool underwent iterative development and testing. Then, cross-sectional content analysis of publicly available documents detailing school food provision (i.e. menus and price lists) was done.

**Setting:**

Primary and secondary schools in Wales, UK.

**Participants:**

In total, 82 canteen menus were sourced online. This comprised local authority catering for primary (*n* 22) and secondary (*n* 19) schools and school-organised catering for primary (*n* 5) and secondary (*n* 36) schools.

**Results:**

Intercoder reliability testing found high agreeability between coders, demonstrating that the tool and data interpretation are reproducible and trustworthy. The FSM allowance is not wholly sufficient for all secondary school pupils to purchase a healthy meal from the school canteen. Moreover, the tool identified that oily fish and wholegrain provision were lacking across many menus.

**Conclusions:**

A valuable tool was created, useful for researchers and other health professionals (i.e. dietitians) who are required to analyse the healthiness of school food provision in line with the latest nutritional requirements. This study provides insight into the current school food and drink landscape pan-Wales.

## Graphical abstract

**Figure:**
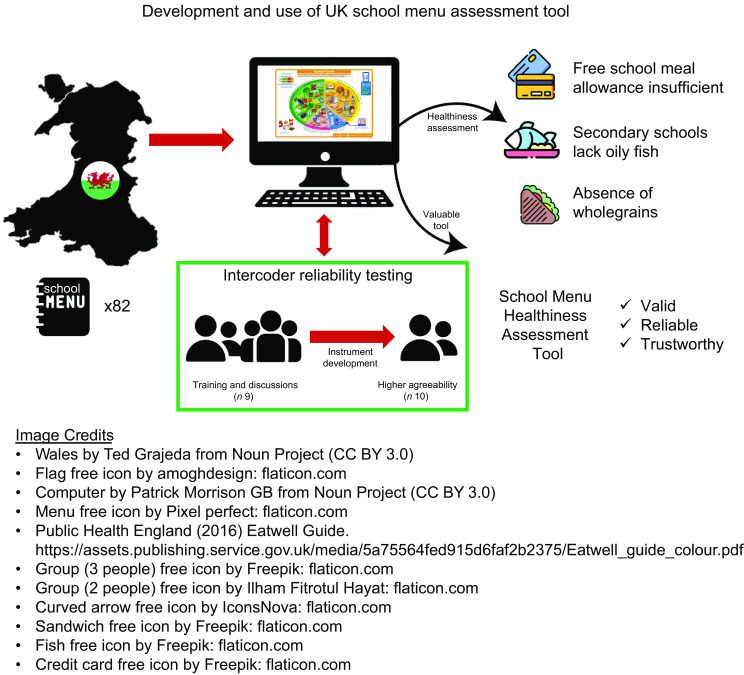

The latest, 2019, National Diet and Nutrition Survey (NDNS) in Wales found that children’s dietary intake is sub-optimal, with 11–18-year-olds averaging 2·7 portions of fruit and vegetables daily, 90 % of children exceeding the free sugars recommendation and 89 % of 4–10-year-olds consuming insufficient fibre^(1)^. Food consumed during the school day equates to 35–40 % of pupils’ dietary intake; thus, the school food environment is a well-established setting for forming healthy eating behaviours^(2)^. Compared to a packed lunch, pupils opting for a school meal typically consume a healthier meal, containing less Na, fat and sugar^(3)^. Consequently, the food and drink available at school play a crucial role in school-aged children’s dietary intake and in establishing healthy eating habits^(2)^.

In acknowledgement of the essential role school canteens play in promoting a healthful diet, School Food Standards (SFS) have been established by all four nations in the UK^(4–7)^. Whilst the Scottish SFS were most recently published in 2020^(6)^, none of the SFS are up-to-date and wholly meet Public Health England’s (PHE) 2016 Eatwell Guide^(8)^ and the latest advice from the Scientific Advisory Committee on Nutrition (SACN) reports published from 2004 to 2023^(9–13)^. In contrast to current nutritional advice, the present Welsh SFS permit bacon daily, do not have a limit on red meat provision, contain no requirements as to the frequency and constitutes of non-dairy proteins and feature no stipulations regarding wholegrain provision^(7)^.

Across the UK, there is a lack of ‘consistent assessment, monitoring or reporting’ of SFS compliance^(14)^. Large-scale systematic reviews evaluating school menu assessment tools indicate that most studies are cross-sectional, taking place in the USA, Brazil or Spain^(15,16)^. Elford *et al.’s* systematic review found seven measurement tools assessing primary school food provision (*n* 35): weighed food protocol (*n* 13), visual observation (*n* 5), menu review (*n* 4), quick menu audit (*n* 4), questionnaire/survey (*n* 3), digital photography (*n* 2) and web-based assessment tool (*n* 1). Notably, many of these tools are resource-intensive (weighing food, observations and photography) or place a burden on the school caterers who may provide socially desirable responses (questionnaires and web-based self-assessment)^(16,17)^. Overall, there is no standardised method to robustly measure school food, and this could contribute to children’s nutritional inadequacy, although a global tool would be difficult to implement considering the heterogeneity of school food provision^(15,17)^.

Aside from the healthiness of school food provision, there is minimal research exploring the food prices within school canteens. Across the UK, pupils are entitled to a free school meal (FSM) allowance if their parents or caregivers earn below a low threshold or are claiming certain benefits^(18)^. Schools are often viewed as a cornerstone of local communities, acting as a designated setting to reduce inequalities in health through the education delivered and school food provision^(19)^. Albeit a difficult task given often inadequate resources and the societal imbalance structuring today’s dietary choices^(20)^, FSM provide access to school food for pupils from lower-income and socio-economically deprived backgrounds. These pupils may lack nutritious food at home, and research shows that they are more likely than non-FSM pupils to opt for the daily ‘meal of the day’^(21)^. Additionally, the Welsh Government established its Universal FSM (UFSM) for all primary school-aged children in September 2024^(18)^.

Research investigating the prices of healthy and unhealthy foods at Australian primary schools found that healthier items were more expensive – especially sandwiches and hot meals^(22)^. Likewise, another study investigated the relative pricing of healthy *v*. less healthy foods across 200 school canteen menus in Australia. The study discovered that 75 % of the primary schools and 57 % of the secondary schools sold the ‘less healthy’ lunch item at a lower price than a ‘healthy’ lunch item^(23)^. A limitation of this research is that the entire pricing structure of menus was not analysed, only the cheapest healthy and unhealthy items. On the whole, there is a dearth of research exploring the pricing structure of school canteen price lists as well as the effect of pricing on school meal uptake and intake.

The purpose of this paper is to outline the development of a new, innovative and valid School Menu Healthiness Assessment Tool (SMHAT) that reflects the latest dietary guidance and is suitable for the quantitative and qualitative analysis of school food provision and, additionally, to use the SMHAT to analyse primary and secondary menus and/or price lists pan-Wales to determine their healthiness and address the literature gap related to whether FSM eligible pupils can afford to access healthy, nutritious food and drink during the school day.

## Methods

### Setting, study design and recruitment

Most schools in Wales are local authority (LA) maintained and consequently are legally obliged to provide a nutritionally balanced ‘meal of the day’, which complies with the *Healthy Eating in Schools (Nutritional Standards and Requirements) Wales Regulations 2013*. Schools must meet these Regulations (aka SFS) whether the food and drink are provided by the LA or alternatively school-organised through a private catering contract^(7)^. In Wales, primary school food is fixed price and characteristically limited to two or three seated main meal options with a dessert. In contrast, the food and drink offering at secondary schools can be vast and always includes sit-down meals along with convenient, on-the-go options. Menus generally run on a 3-week rotational cycle and are changed approximately twice per annum. The FSM allowance for primary and secondary pupils varies across each LA in Wales.

**Figure 1.**
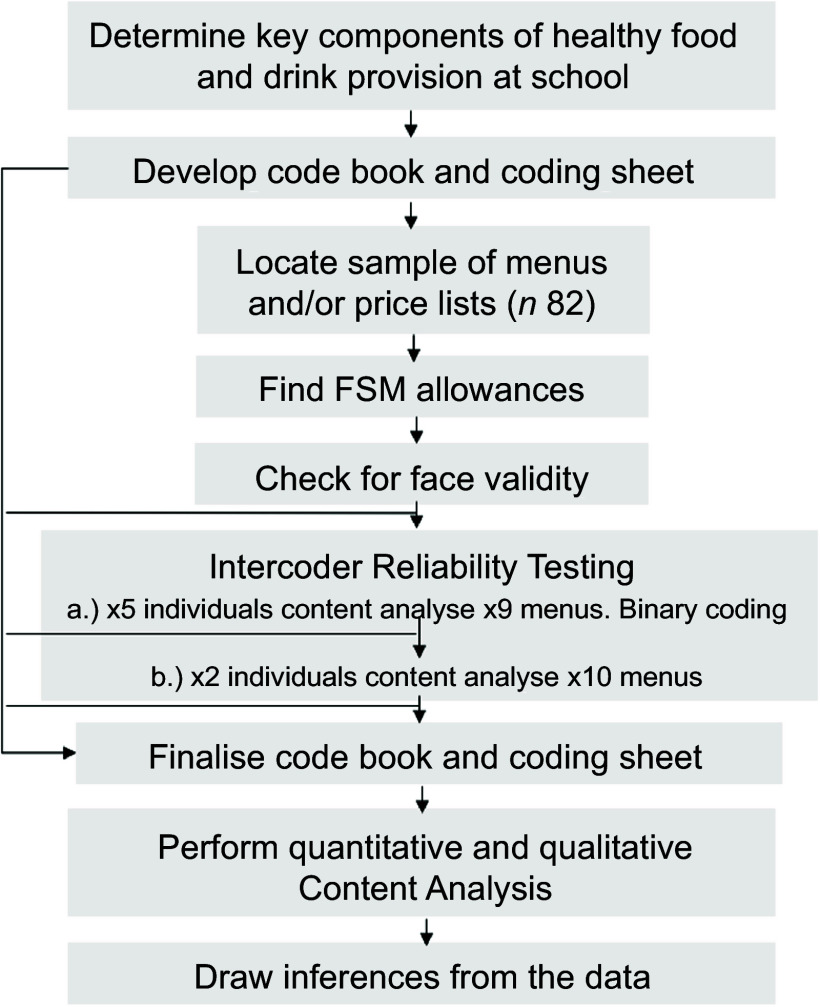
Flow chart showing the development of the SMHAT. SMHAT, School Menu Healthiness Assessment Tool.

The aim of the present research study was to first develop the SMHAT and then to quantitatively and qualitatively analyse school food provision pan-Wales to determine the extent to which it meets the latest healthy eating government recommendations for school-aged children^(8–13)^ in addition to its affordability (Fig. 1). The sampling target was to obtain a total population sample of every primary and secondary school menu (*n* 104) and/or price list. A cross-sectional approach was taken, collating and analysing school menus and price lists during autumn 2023. All twenty-two LA websites were searched for an up-to-date FSM allowance as well as a primary and secondary school menu (*n* 44 total). In addition, sixty schools in Wales organise their own catering. Therefore, the total population sample would be 104.

Menus were obtained from either LA or school websites (*n* 75), and seven LAs were contacted via email, online messaging or using a Freedom of Information request to ask for the menu if it was not freely available. Twenty-two menus could not be located, meaning that eighty-two menus and/or price lists were analysed in the present study. Menus written in the Welsh language were translated for the English-speaking research team via an online translation website. All menus were copied and pasted or screenshot and then saved in a PDF format for uniformity. Most menus mentioned ‘autumn 2023’, but if not, it was assumed that the menus available online were in current use by the school or LA^(24)^.

### The School Menu Healthiness Assessment Tool

There are no standardised or internationally recognised methods for measuring the school food environment and analysing menus^(17)^. Subsequently, a quick menu auditing tool, the SMHAT, was developed to assess school menu healthiness based on applicable sections of the Welsh SFS, encompassing the latest dietary guidelines modified for primary and secondary school-aged children as criterion^(8–13)^. Experienced nutritionists working within public health worked alongside nutrition researchers to decide and operationalise pertinent categories and items as well as the scoring system thresholds. The categories included: breakfast; break time; fruit and vegetables; meat, fish and alternatives; starchy carbohydrates; dairy products and alternatives; oils and spreads; high in fat, salt and sugar (HFSS) foods; prices; and general observations. Several of these categories were derived directly from the Eatwell Guide^(8)^. The majority of schools in Wales provide breakfast and break time food; thus, they were included in the analysis to provide a better indication of the overall healthiness of food and drink provision across the school day. The SMHAT ‘items’ are food-based criteria rather than nutrients, meaning no secondary nutritional analysis is required.

Both quantitative and qualitative content analyses (CA) were utilised to comprehensively audit school food and drink healthiness^(25,26)^. One tool was developed for primary school-aged children and another for secondary school-aged adolescents, reflecting the different dietary requirements for these age groups. The CA tools were established prior to analysis and consist of two parts, the code book (Microsoft Word document) and the coding sheet (Microsoft Excel workbook)^(25)^. The scoring methodology is explained in online supplementary material, Supporting Information Table A and B. In general, ‘A’ was assigned for an item that was absent (not mentioned), ‘1’ for a category where the menu items met the SMHAT criteria and ‘0’ if they were not met. Incremental scores of 0·2–1·0 were possible for categories such as vegetables where the SFS indicated ‘at least one portion per day’ given the school week has 5 d.

Each week of the menu cycle was coded separately according to the code book, and the total score was divided by the number of weeks to provide a comparable healthiness score for each school food provision analysed. The higher the score, the closer the school food provision is to the ascribed ideal healthiness standard, with a maximum primary school food provision score of 22 and 28 being the highest score for secondary schools. The SMHAT also permits qualitative notetaking and thematic analysis as wholly quantitative CA can be too reductionist and fail to show the intricacies within the dataset^(25,26)^. The qualitative comment boxes allow coders to make note of the pricing, item descriptions and the variety on offer. The average (*M*) cost of menu items was calculated using Microsoft Excel.

### Intercoder reliability testing

The face validity of the tool was initially checked by public health nutritionists and their feedback informed amendments to the SMHAT. Intercoder reliability (ICR) is widely recognised to be an essential component of credible CA studies^(28,29)^. Hence, ICR testing was first performed with five coders analysing four primary menus and five secondary menus (see online supplementary material, Supporting Information C). Following in-depth SMHAT training (delivered by X.X.), coders independently analysed one menu. Discrepancies in the coding were discussed, and a consensus was formed before coders independently coded another eight menus using the tool. Feedback and comments garnered from the scoring, sd and ICR testing indicated which items and categories were problematic and causing intercoder disagreements^(25,28,29)^. As ‘A’ is a non-numerical rating, binary coding was conducted to calculate the level of agreement across multiple coders.

Inconsistent scoring and coder feedback of ambiguity, for instance, scoring red meat, led to modification of the code book to increase the clarity of how certain items must be scored. Next, two coders (X.X. and X.X.) completed CA for an additional ten menus. Statistical analysis was performed using Statistical Package for the Social Sciences version 29 (SPSS Inc.). SPSS was utilised to calculate Cohen’s kappa (κ) based on the two coders’ CA scoring of three primary and seven secondary school menus. A larger proportion of secondary school menus were assessed as these made up a greater proportion of the sample, and the first stage of ICR testing showed the greatest discrepancies in the scoring for secondary school food provision.

McHugh’s values for health research were adopted for interpreting the interrater reliability of κ: scores of 0·60–0·79 implied a moderate level of agreement, 0·80–0·90 indicated a strong level of agreement and scores over 0·90 showed an almost perfect level of agreement^(30)^. Discussion following the second stage of ICR testing allowed any ambiguity to be clarified, and again, minor revisions to the code book were made. The final code book and coding sheet were used by one coder (X.X.) to analyse all eighty-two school food menus and/or price lists pan-Wales.

## Results

### The two stages of intercoder reliability testing

Statistical testing and descriptive statistics were used to calculate the validity of the SMHAT. As aforementioned, the first stage of the ICR testing involved five coders analysing a total of nine menus. Primary school menu scoring deviated less from complete agreement than the secondary menu and/or price list CA scores. Identification of at least one wholegrain carbohydrate a week had a low *sd* on average (Primary 0·248; Secondary 0·249) *v*. the red meat rating which was the highest level of average *sd* for both menu subgroups (Primary 0·480; Secondary 0·485). There was a higher level of variation for items where the coders interpreted the code book differently (see online supplementary material, Supporting Information C). Following amendments to the SMHAT, the second ICR testing with two coders featured Cohen’s κ, and again, levels of agreeability were higher for the primary menus (Table 1). According to McHugh’s interpretation values for level of agreement, four were ‘perfect’, three were ‘strong’ and three showed a ‘moderate’ level of agreement^(29)^.

**Table 1.**
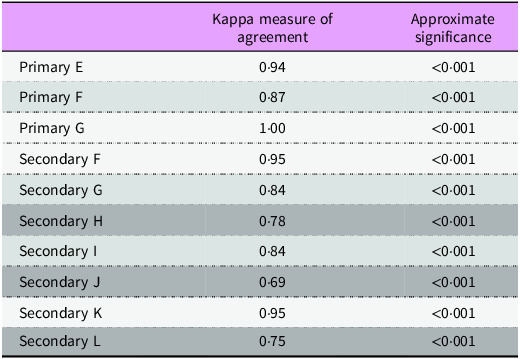
The second stage of ICR testing and the κ measure of agreement between two coders analysing three primary and seven secondary menus

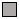
Moderate level of agreement.

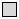
Strong level of agreement.

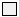
Almost perfect level of agreement.

ICR, intercoder reliability.

### Sample

Locating menus and/or price lists from 79 % (*n* 82) of the total population (*n* 104) indicates that the sample is highly representative of school food provision pan-Wales. Two to seven menus and/or price lists from each LA were analysed, and the total healthiness scores varied widely (Table 2). Two secondary LA and three secondary school-organised catering did not have a menu cycle available for analysis, meaning that only price lists could be coded in lieu of a comprehensive menu cycle. Consequently, these healthiness scores were significantly lower (2·5–6·5) and considered anomalies, so they were omitted from the average score calculation to not skew the results. The largest proportion of food provision analysed was derived from secondary schools with school-organised catering (44 %). Primary LA catering scored the most highly overall and on average.

**Table 2.** Overall number of menus analysed per menu subgroup, range of healthiness scores and SD

Menu subgroup	Included in analysis	Lowest healthiness score omitting anomalies	Average healthiness score omitting anomalies	Highest healthiness score	sd	Maximum healthiness score attainable
Primary LA	22	8·333	12·670	16·500	1·782	22
Secondary LA	19	7·33	10·097	12·717	2·371	28
Primary school-organised	5	7·497	9·942	13·997	2·669	22
Secondary school-organised	36	6·997	9·866	13·820	2·588	28

LA, local authority.

### Healthiness of food and drink provision

#### Breakfast and break time

Sixty (73 %) did not have any information about breakfast provision. Of the twenty-four secondary LA or school-organised break time provision analysed, 92 % had fruit available and 63 % provided bacon daily. Additionally, many offered traditionally lunchtime options at break time: pasta, sandwiches, paninis, soup, ramen noodles and baguettes.

#### Fruit and vegetables

Primary schools were significantly more likely to fulfil the SMHAT fruit and vegetable healthiness criteria (96 %) compared to secondary menus (75 %).

#### Meat, fish and alternatives

The fulfilment of this SMHAT healthiness criteria differed by menu subgroup (Table 3). With the exception of vegan dairy alternatives and vegetarian or vegan meat protein alternatives, primary menus met this criterion more consistently than the secondary menus. Fish and oily fish were a rare occurrence on the secondary menus analysed. The majority of schools met the meat cut provision due to an abundance of chicken-based dishes. However, the predominance of chicken resulted in low red meat scores across most menus, with only four having too much red meat. Some secondary menus had meat products or processed meat (i.e. chicken popcorn and bacon) available daily, resulting in a substantially lower score for this item than the primary menus. However, secondary LA scored most highly in their non-dairy, non-meat protein provision and a large number of innovative dishes were detected: loaded jackfruit skin on fries, veggie dawgs, mango curry and Buddha bowls.

**Table 3.** Fulfilment of the SMHAT criteria for meat, fish and alternatives provision by menu subgroup

	Primary authority menus (*n* 22)	Secondary local authority menus (*n* 19)	Primary school-organised menus (*n* 5)	Secondary school-organised menus (*n* 36)
Scored ‘1’ for fish provision	100·0 % (*n* 22)	26·3 % (*n* 5)	100 % (*n* 5)	22·2 % (*n* 8)
Scored ‘1’ for oily fish provision	77·3 % (*n* 17)	36·8 % (*n* 7)	60 % (*n* 3)	22·2 % (*n* 8)
Scored ‘1’ for meat cut provision	95·5 % (*n* 21)	73·7 % (*n* 14)	100·0 % (*n* 5)	88·9 % (*n* 32)
Scored ‘1’ for red meat provision	22·7 % (*n* 5)	5·3 % (*n* 1)	20·0 % (*n* 1)	5·6 % (*n* 2)
Scored ‘1’ for meat products or processed meat	95·5 % (*n* 21)	78·9 % (*n* 15)	100 % (*n* 5)	72·2 % (*n* 26)
Scored ‘1’ for non-dairy, non-meat protein provision	59·1 % (*n* 13)	68·4 % (*n* 13)	0	58·3 % (*n* 21)

SMHAT, School Menu Healthiness Assessment Tool.

#### Starches and wholegrains

Fourteen menus (17 %) had at least 1 d whereby pupils had no starchy carbohydrate alternative to potatoes. The choice of carbohydrates in these cases were often chips, potato wedges or a baked potato. Menu offerings with bread, sandwiches, a pasta bar or noodles scored more favourably in the SMHAT. Occurrence of wholegrain provision meeting the SMHAT criteria ranged from 17 % (*n* 3) secondary LA menus to 41 % (*n* 9) within primary LA menus.

#### Dairy

Only six menus (7 %) specified semi-skimmed or skimmed milk. A further thirty-three (40 %) had some form of milk available. No menus or price lists featured low-sugar yogurts, but fifty-three (65 %) had yogurts available. The highest incidence of yogurts was observed on LA primary menus (73 %, *n* 16) as these were a standard dessert. Only one of the menus analysed – a secondary LA menu – had soya milk on the price list. Aside from this, there was no mention of any non-dairy milk alternatives.

#### High in fat, salt and sugar food and drink

Confectionery or savoury snacks (i.e. crisps) which did not fulfil the SMHAT healthiness criteria were identified at three (8 %) of the secondary schools with school-organised catering. Elsewhere, savoury snacks (i.e. cheese and biscuits, oatcakes) adhered to the SMHAT code book. The vast majority provided HFSS sweet snacks (i.e. biscuits, cookies, flapjacks, muffins and traybake cakes). The free sugar in secondary school drinks proved impossible to rate for fifty (91 %) menus and/or price lists due to a lack of information provided. Salt was not stated to be freely available across any of the menus analysed, as per the current Welsh SFS.

### Free school meal allowance

FSM allowance varies by LA in Wales, and across the twenty-two LAs, the allowance for 73 % (*n* 16) primaries and 59 % (*n* 13) of secondaries were located online. In LA where FSM are provided to families with low income, this varied from £2·20 to £3·00 (*M* £2·52, *sd* 0·24). The FSM allowance for secondary school food varied from £2·40 to £3·10 (*M* £2·70, *sd* 0·23).

Price lists were not always available; however, one LA FSM allowance prohibited these secondary school pupils from purchasing the meal deal (main meal and drink) at two schools with school-organised catering as there was a £0·15 price disparity. Likewise, in another LA, a school sold meal deals at £0·06 higher than the FSM allowance. Conversely, a different LA FSM allowance proved plentiful as three school-organised menus in the LA had a meal deal price that was substantially (£0·65, £1·10 and £1·25) lower than the FSM allowance. In other LAs, meal deals were priced merely £0·20 below the FSM allowance, possibly allowing pupils to purchase an inexpensive condiment sachet – often the only item affordable (*M* £0·11).

No obvious pattern was observed, as a higher FSM allowance did not necessarily equate to higher school food prices or vice versa. Welsh pupils dependent on the FSM allowance for their school food and drink were outpriced by several options, which would restrict their choices, as in some cases bacon baguettes, paninis, salads and filled baked potatoes exceeded their respective FSM allowance.

Secondary school price lists were analysed and fifty-five HFSS snack prices were identified, averaging £0·92 (*sd* 0·17). A slice of toast (*n* 22) most commonly cost £0·35 (*M* £0·38, *sd* 0·07). A portion of vegetables (*n* 12) averaged £0·54 (*sd* 0·13), more expensive than a whole fruit piece (*n* 18) averaging £0·49 (*sd* 0·11). Fruit salad pots (*n* 16) were considerably more expensive, costing £0·96 on average (*sd* 0·25).

## Discussion

### Healthiness of food and drink provision

Presently, LA maintained schools must only comply with the Welsh SFS^(7)^, whilst the SMHAT has heightened criteria for healthy school food and drink provision. Accordingly, it was not expected that menus pan-Wales would score highly in their fulfilment of the SMHAT criteria. Primary schools had higher SMHAT scores compared to secondary schools, and this was in accordance with the literature, which indicated LA staff believed primary schools to be more SFS compliant as there are fewer options and an increased emphasis on a nutritious lunch^(31)^. New-found autonomy at secondary schools can cause adolescents to eat less healthy options. Focus groups with Welsh adolescents discovered that they express a strong preference for portable foods which can be consumed on-the-go^(32)^. This preference may be attributed to the typical secondary school food environment having long queues, a short lunch break and being chaotic^(32,33)^.

School canteen menus typically adhere to adolescents’ desire for on-the-go foods by providing processed meats and snackable items devoid of vegetables. It is acknowledged that the skills and passion of canteen staff impact their ability to incorporate nutritious dietary components into desirable, on-the-go food – particularly at secondary schools where the need for convenience is greater. Taking the wider school food system into account is important when instigating change to menus^(33)^.

Two areas whereby healthiness scores were particularly low included oily fish provision and wholegrains. The SMHAT used the existing Welsh SFS for oily fish (twice over a 4-week period)^(7)^, yet found the majority of secondary schools did not meet the recommended levels. Fish and oily fish are renown as being unpopular and intake is low amongst Welsh children^(1)^, resultantly catering staff may serve this less frequently to minimise food wastage^(34)^. This is problematic as serving fish at school lunchtimes may contribute to increased *n*-3 and *n*-6 consumption whilst setting ‘the pattern for healthy habits in adult life’^(35)^. Despite this knowledge, the school canteen must balance their financial viability with students’ preferences and custom^(33)^.

The second area of concern was the lack of wholegrains pan-Wales. Presently, only the English SFS mention wholegrains provision on a weekly basis despite the known association between wholegrain consumption and a lowered risk of some cancers and diseases^(4,11)^. Although the current Welsh SFS^(7)^ does not feature wholegrain recommendations, the SMHAT was designed to rate menus which featured this item weekly with a top score of ‘1’. A systematic review of public health interventions aiming to increase wholegrain intake found that the captive audience in an educational setting (i.e. schools and colleges) should be taken advantage of so that healthy wholegrain consumption can be instilled at a young age^(36)^. School caterers and policymakers ought to take this into consideration.

Several items within the SMHAT directly relate to the current SFS in Wales^(7)^; nonetheless, instances were observed where food provision did not fulfil the SMHAT criteria. Hence, when the SFS are next reviewed, the latest dietary guidance and recommendations should be considered as well as the ability of schools to meet current SFS. It is recommended that the prevailing SFS is not to be used as a baseline for policymakers. Instead, evaluation could be undertaken by incorporating the SMHAT into any new policy developments. When SFS are developed and implemented, the local context, locally available foods and dietary customs must be taken into account^(37^ ^,38)^.

### The affordability of healthy food and drink

The price of fruit (£0·54) was substantially lower than HFSS sweet snacks (£0·92) which was surprising as an Australian study found that healthy options were on average $1·00 AUD more expensive than unhealthy options in school canteens^(23)^. The prices obtained in this study build on existing research, but to date there is no holistic, comprehensive price analysis of all healthy *v*. unhealthy options in school canteens. This is in part due to difficulties amassing the price lists from a substantial sample^(22,23)^. Moreover, little is understood about how school food pricing affects pupils’ decision-making across all menu categories^(22)^.

As aforementioned, schools are well positioned to reduce inequalities in children’s health and the food and drink provision is particularly crucial for those from a lower socio-economic background^(2,19,38)^. As the ‘meal of the day’ needs to be nutritionally analysed according to the Welsh SFS, it tends to be one of the most nutritionally balanced options on the school menu^(7)^. Yet, the present study ascertained that some secondary school pupils entitled to a FSM allowance cannot always afford the meal of the day. This finding was in concordance with prior research which also discovered that the FSM allowance restricts what items can be purchased and does not guarantee the purchasing cost of a hot meal for secondary school pupils^(38,39)^.

Even if a secondary school pupil is able to purchase a meal with their FSM allowance, across many schools they would not have any spare allowance to purchase a breakfast or break time item. Further, breakfast skipping is prevalent amongst adolescents, and late lunch timetabling may result in these pupils opting for a break time item and subsequently only being able to afford a snack at lunch. To improve academic performance and the nutritional status of school-aged children, it is paramount pupils are adequately fed across the school day – not only at lunchtime^(40)^.

This study posits that the secondary FSM allowance has a duty to adequately cover healthy, nutritious food from the school canteen. More research is needed elsewhere in the UK to assess how common unaffordability of school food for FSM pupils is. Generally, studies have found ‘price is seldom considered in healthy school food policies’ as profitability is prioritised above pupil health^(17)^. If the practice is widespread, then policymakers must work to either increase the FSM allowance or reduce the prices in the school canteen. It is acknowledged that food pricing in the school canteen is partially determined by the cost of labour and ingredients. Yet, pricing strategies could be applied to encourage secondary school-aged adolescents to make healthier purchasing choices without impeding on the school’s revenue^(22)^. Regarding primary school nutrition, UFSM trials in England and Scotland have resulted in increases in school food uptake, so a similar effect may be observed in Wales^(18)^. In line with the UK’s health agenda and commitment to improving children’s health, school food provision must take into account the complexities of consumption preferences, catering costs and time constraints^(14,32)^.

### The School Menu Healthiness Assessment Tool

A systematic review has found a lack of methodology or tools for evaluating school food menus^(15)^. Operationalising the constituents of nutritious school food and drink provision for primary and secondary school-aged children determined the construct of the SMHAT. Collaboration between nutrition researchers and public health nutritionist has resulted in a valid yet comprehensive quick menu auditing tool. Compared to the acquisition and use of expensive nutritional analysis software which requires considerable training, the SMHAT can be simply undertaken using a spreadsheet and web browser, so it is accessible to a wider pool of users^(16,17)^. Moreover, the SMHAT takes less time and is resource-intensive compared to in-person food assessment tools such as observation or weighed food protocols and may be considered more comprehensive than existing tools measuring school food and drink provision^(16,22–24,41)^. Incorporating spaces for qualitative notetaking within the SMHAT prevents the analysis becoming too number-focused and reductionist^(26)^. Thematically analysing the descriptive language used together with the various menu options gives an in-depth understanding of the current school food and drink landscape pan-Wales^(27)^.

Extant school menu auditing tools heavily rely on self-reported assessment; yet there is no incentive for schools to accurately report their provision^(16,17,41)^. A key advantage of the quick menu auditing tool was that it eliminated the need to obtain supplementary information from schools. Furthermore, studies using a quick menu auditing tool typically analyse 53–168 schools, making it well suited for the data collection pan-Wales^(16)^. Compared to previous school menu auditing studies, the sample size obtained was largely representative, equalling 79 % of the total population sample. In comparison, another school menu auditing study was only able to locate 49 % of their predetermined sample size of menus online^(24)^.

The final code book and coding sheet underwent face validity testing and two stages of ICR testing to validate the tool. Establishing the ICR for the CA scoring provided rigour to the study, resulting in credible data interpretation and a valid quick menu auditing tool^(28,29)^. Categories whereby there were a higher level of discrepancies (i.e. meat, fish and alternatives) are not necessarily a weakness of the SMHAT, but instead expose the poor comprehension of menus, which will also prove an issue for pupils and parents. Primary school menus exhibited a level of higher agreeability between coders in the ICR testing. This may be attributable to the variety of options^(32)^ and lack of clarity across secondary school menus and/or price lists. High ICR testing scores indicated high agreeability and imply that the study findings are reproducible and trustworthy^(29,30)^. Accordingly, the higher the ICR score, the more confidence there is in the interchangeability of the scores given by one coder and another coder.

Analysing most of the school food and drink provision in Wales provided a clear indication of how certain items differ between menu subgroup and the variety of food on offer. The ability to quantify each menu and/or price list healthiness score offered insight into menus which are clear outliers. Moreover, a systematic review has highlighted a dearth of literature concerning school meal evaluation, with many failing to assess the validity or reliability of utilised tools and several published articles not written in the English language^(17)^. It is expected that the SMHAT is a worthy resource for calculating the healthiness of school food and drink provision UK-wide. This addresses the gap in the academic literature pertaining to school food and drink in Wales as most studies exploring the impact of SFS in the UK are limited to the English SFS^(14,31)^.

The study has some limitations. First, the menus and/or price lists included in this analysis were located online for the CA. Nonetheless, it must be recognised that menus are liable to change on a day-to-day basis depending on the availability of ingredients or other external factors. Second, incomplete menus and information to rate meant that data analysis was limited for five secondary schools or LAs which lacked a menu cycle. Solely analysing price lists meant that these instances had a lower, incomplete healthiness score. Further, drink menus were absent across many schools, and those with a comprehensive drinks menu often lacked brand names or quantities, meaning certain items were unrateable in the coding sheet. The lack of access to the full range of menu offerings limited the extensiveness of the analysis. Of course, the aforementioned limitations could be addressed via school canteen observations, but auditing menus has been considered a sufficient, if limited means of measuring the healthiness of school food provision in the literature^(24,41)^. Lastly, the SMHAT is not infallible and scores may slightly differ depending on the coder’s interpretation of the code book.

Strengths of this study include the stringent tool development process and inclusion of ICR testing to improve the rigour of the SMHAT. Collating menus online avoided participant interaction or selection bias which can be confounding variables in self-reported canteen analysis^(16,41)^. Of course, the tool is adaptable and may be changed and updated to reflect the new SFS.

## Conclusions

In conclusion, the present paper introduces the SMHAT, an innovative tool for evaluating the food and drink provision at primary and secondary schools in the UK. It allows the calculation of school menu and/or price lists healthiness against the most up-to-date governmental healthy eating guidance without the requirement to purchase software or undertake a detailed nutritional analysis. The tool has implications for both research and practice, providing an effective tool for both academic researchers and public health professionals. Further, the paper provides insight into the current healthiness landscape of school food and drink provision pan-Wales. This will prove useful for policymakers who are seeking to update the SFS. Although this study investigated whether the FSM allowance is sufficient in allowing pupils to access healthy food across the school day, more research is needed in this area.

## Supporting information

Gilmour and Fairchild supplementary material 1 Gilmour and Fairchild supplementary material

Gilmour and Fairchild supplementary material 2 Gilmour and Fairchild supplementary material

## Supplementary material

For supplementary material accompanying this paper visit https://doi.org/10.1017/S1368980025000047
