# Supplementary material for: Developing and using a School Menu Healthiness Assessment Tool to analyse school food provision in Wales
Source: Public Health Nutr. 2025 Jan 10;28(1):e36. doi: 10.1017/S1368980025000047 (PMC11822622; doi:10.1017/S1368980025000047)
Supplement: Gilmour and Fairchild supplementary material 1 — Gilmour and Fairchild supplementary material [file S1368980025000047sup001.docx]

A. Primary school food provision code book

|  | **Description** | **Coding** |
| --- | --- | --- |
| **Breakfast** | i. Menu inclusive of permitted categories | ‘A’ for the absence of any school breakfast menu or price list. Some schools may have a price list or additional information. It should not be assumed that all these foods are available at breakfast, unless it is explicitly stated – so an ‘A’ should be given. ‘1’ rating for menus featuring only foods in the four categories permitted by the *Regulations* (see Justification / Explanation column), regardless of how many categories are fulfilled. Menus containing a non-permitted food or drink item will be rated ‘0.’ If bacon is on the breakfast menu, this breakfast category should be rated ‘0.’ |
| **Break time** | i. Fruit and/or vegetables must be available | An ‘A’ will be given where no information is provided about break time provision. Otherwise, ‘0’ if there is break time provision available to rate and there are no fruit and/or vegetables available and ‘1’ if these are available. |
|  | ii. No cakes or biscuits permitted | If no information is provided, an ‘A’ will be given. If cakes and biscuits are on the menu, a ‘0’ will be given. Bagels, pikelets, crumpets and teacakes do not fall under the ‘cakes and biscuits’ break time category. It is assumed that tea cakes are not *Tunnock’s* teacakes (biscuit and marshmallow), instead tea cakes are a sweet bread made with dried fruit and are permitted during break time. If cakes and biscuits are on the price list, it should not necessarily be assumed that these are available at break time. |
|  | iii. No confectionery or savoury snacks may be provided, except crackers, water biscuits and oatcakes | ‘A’ will be given where there is no information provided. If confectionery and/or savoury snacks are on the menu, a ‘0’ will be given. If bacon is on the menu, a ‘0’ will be given for this criterion. |
|  | iv. Notes | This will be purely qualitative notes. The coder should write if there are ‘lunchtime’ options available at break time – e.g., pasta, sandwiches, wraps and baguettes. It is anticipated that primary schools do not have a break time menu. |
| **Fruit and vegetables** | i. At least one portion of vegetables or salad provided daily | Provision is to be calculated per day (i.e., if there are four portions on one day, this will only total ‘one’ portion for that day as only one is required daily for primary school lunches). If the menu generically states, ‘seasonal vegetables’ then it must be assumed that there is sufficient variety and be rated ‘1.’ Otherwise, an incremental decimal of 0.2 from 0 to 1 should be given (5 days in a week) based on the variety available over the 5-day week. E.g., if only ‘peas’ are on the menu five days a week, the rating would be ‘0.2.’ Those with only two different vegetables across the week would receive a ‘0.4’ rating. Menus with a good variety of vegetables across the week, differing daily would receive a ‘1.’ All menus should feature vegetables, so a ‘0’ will be given if these are not seen. The scoring for this criterion is dependent on the variety available across the menu. |
|  | ii. At least one portion of fruit, fruit salad or fruit juice provided daily | If there is no mention of fruit on the menu or price list, an ‘A’ should be given. Provision is to be calculated per day (i.e., if there are multiple portions on one day, this will total ‘one’ portion for that day. If the menu generically says that fruit is available, it must be assumed that there is variety and a ‘1’ will be given. Otherwise, an incremental decimal of 0.2 from 0 to 1 is to be given (5 days in a week) based on the variety available over the five-day school week. Examples as above. If fruit is tinned, make note of this. |
|  | iii. A fruit-based dessert (containing at least 40g raw fruit ingredients) twice a week | If a dessert appears heavily fruit-based, i.e., apple pie or fruit tart then it is assumed that the raw fruit quota is met. Weeks with no fruit-based desserts will receive a ‘0,’ a week with one fruit-based dessert will receive a ‘0.5’ and those with two or more will receive ‘1.’ If there is no mention of desserts on the menu, this item will be coded ‘A.’ The coder should read the menu carefully and do searches for ‘apple’ and ‘crumble’ etc. to ensure these are not missed. Fruit jelly, Eve’s pudding, carrot cake and Welsh cakes contain enough fruit to be counted as a ‘fruit-based dessert.’ However, cheesecake, a lemon drizzle or orange zest cake do not contain enough raw fruit to count as a ‘fruit-based dessert.’ It should not be assumed that ‘homemade desserts’ are fruit-based and meet the criteria. Fruit wedges as part of a primary school dessert menu will count as a ‘fruit-based’ dessert. The coder should write a comment if any assumptions are made |
|  | iv. Fruit or vegetable juice combined with water should only be available at breakfast or lunchtime | ‘A’ will be given where there is no information provided. Otherwise, ‘0’ if the juice is available at all times (i.e., break time as well) and ‘1’ if available at breakfast and lunch. |
| **Meat, fish and alternatives** | i. A portion of fish at least once each week | Provision is to be calculated per day (i.e., if there are multiple portions on one day, this will total ‘one’ portion). The BBC *Good Food* website must be used to check menu ingredients. Menu weeks with no mention of fish will receive a ‘0’ and those with one portion of fish will receive a ‘1.’ Information that is not in the menu cycle should also be included in the count. For example, if tuna is available (i.e., in a baguette or jacket potato), the menu should be rated ‘1.’ If fish type is not specified, it should be assumed that the fish is non-oily. |
|  | ii. A portion of oily fish at least twice during any four-week period | Menu cycles with no oily fish at all will receive a ‘0’ and those with two portions per four weeks will receive a ‘1.’ Menus with one portion per four weeks will receive a ‘0.5.’ To meet the primary school menu criteria, oily fish must be available once every two-week cycle or twice per three-week cycle. |
|  | iii. Meat cuts on at least two days a week (not including luncheon meat – i.e., sliced ham) | Meat cuts include: any meat or poultry including joints, cooked sliced meat; bacon or mince (ground) meat. Provision is to be calculated per day (i.e., if there are multiple portions on one day, this will total ‘one’ portion). The BBC *Good Food* website must be used to check menu ingredients. Menu weeks with no meat cuts will receive a ‘0,’ menus with meat cuts on one day will receive a ‘0.5’ and those with two or more days featuring meat cuts will receive ‘1.’ Menu items coded as a ‘meat cut’ can include red meat cuts – these will be coded twice. |
|  | iv. Red meat a maximum of two days each week | Provision is to be calculated per day (i.e., if there are multiple portions on one day, this will total ‘one’ portion). For example, if there are four types of red meat dish available on a single day, this would count as ‘one portion’ as pupils would only consume one per day. For rating primary school menus, red meat provision must be across two different days to receive the top mark of ‘1’. The BBC *Good Food* website must be used to check menu ingredients (e.g., Frikadelle is a red meat item). Menus with no red meat will receive a ‘0,’ those with one portion of red meat will receive a ‘0.5’ and those with two or more portions will receive a ‘1.’ This criterion is focused on lunchtime, so if there is red meat available daily at break time or breakfast, that should not be taken into account for this score. If there are different options given for a roast dinner (i.e., turkey, pork, chicken or gammon), it should be assumed that red meat is offered and this would count as a portion of red meat (one out of a limit of two across the week). Burgers that do not specify the meat are assumed to be beef (red meat) and are not to be classified as a ‘processed meat’ or ‘meat product.’ It should be assumed that burritos, lasagne and meatballs contain red meat. Lastly, sausages are a ‘processed meat’ and should not be counted as a ‘red meat.’ |
|  | v. No more than two 'meat products' or ‘processed meat’ portions each week | Provision is to be calculated per day (i.e., if there are multiple portions on one day, this will total ‘one’ portion). The BBC *Good Food* website must be used to check menu ingredients. Menus featuring meat products on two days or fewer will receive a ‘1’ and those with ‘processed meat’ or ‘meat products’ on three or more days will receive a ‘0.’ Sausages are a ‘processed meat’ and should not be counted as a ‘red meat.’ Ham, gammon and bacon are counted as a ‘red meat’ and a ‘processed meat.’ Chicken goujons are a ‘meat product’ or ‘processed meat,’ unless it is specified that these are chicken breast or ‘homemade’ – in which case they would be classified as a ‘meat cut.’ Pepperoni and salami (i.e., as a pizza topping) are counted as a processed meat, but the quantity is too small to also be counted as a ‘red meat.’ Non-meat, non-dairy options are not a ‘processed meat’ or ‘meat product.’ |
|  | vi. For vegetarians / vegans, a portion of non-dairy protein three or more days a week | Provision is to be calculated per day (i.e., if there are multiple portions on one day, this will total ‘one’ portion). It is important to include the price list non-meat, non-dairy protein sources in the count – the extent to which the price list is used should be commented upon by the coder. The BBC *Good Food* website should be used to assess whether a menu item is a source of protein: i.e., the BBC *Good Food* vegetarian lasagne has vegetables rather than pulses or soya protein, so it is assumed that vegetarian lasagnes are not a protein source; a vegetarian curry is assumed to contain protein (likely pulses or beans); quesadillas contain cheese so are not a ‘non-dairy protein source.’ Menus without any non-meat, non-dairy protein will be rated ‘0.’ Those with one in a week will be rated ‘0.33’ and those with two will be rated ‘0.66,’ with prevalence of three or more days being rated ‘1.’ Comments should be made if the non-meat, non-dairy options appear to be largely UPFs. If the only suitable option is baked beans and a jacket potato available daily, this menu would be rated ‘0.33’ due to a lack of variety. |
| **Potatoes, bread, rice, pasta and other starchy carbohydrates** | i. A portion of potato or potato product cooked in oil a maximum of twice a week | Menus surpassing this and featuring three or more portions of potato or potato product cooked in oil will receive a ‘0.’ Menus with two or fewer portions of potato or potato product cooked in oil will receive a ‘1.’ Unless otherwise specified, it will always be assumed that roast dinners contain roast potatoes which are cooked in oil. Diced potatoes, wedges and hasselback potatoes are assumed to be cooked in some oil, unless it is stated these are oven-baked (purchased frozen chips and wedges are likely to be pre-cooked in some oil). If the menu description of potatoes is vague, i.e., ‘potato’ or ‘Cooks choice potatoes’ then it should be assumed that these are not cooked in oil as there are numerous other ways in which potatoes can be cooked. |
|  | ii. One or more wholegrain starchy food each week | Menus with no wholegrain starchy foods will be rated ‘0’ and those with at least one wholegrain starchy food across that week will be rated ‘1.’ If there is no mention of wholegrains or this cannot be deciphered, the rating will be an ‘A.’ 50:50 bread may be counted as a wholegrain starchy food, but a note should be written if this is the case. |
|  | iii. At least one portion of starchy carbohydrates (excluding potatoes) must be provided daily | Menus without any starchy carbohydrate alternative to potatoes will receive a ‘0.’ Those with one or more alternatives will receive a ‘1.’ |
| **Dairy and alternatives** | i. Provided milk must be semi-skimmed or skimmed | If there is no mention of milk, an ‘A’ rating will be given and if semi-skimmed or skimmed milk is specified, a ‘1’ rating will be given. If milk is provided but there is no indication as to whether it is semi-skimmed or skimmed, the rating will be ‘0.5.’ Milk can include milkshakes as it is presumed that there is a high volume of dairy in the drink. Any milk, even if only available once or twice a week should be rated either ‘0.5’ or ‘1.’ The coder may write a comment about frequency of provision. |
|  | ii. Soya, rice or oat milk must be unsweetened (<5% added sugars) and contain added calcium | No mention of non-dairy milk alternatives will be rated an ‘A.’ If the milks are provided, but there is no description of sweeteners or fortification, a ‘0’ will be given. A ‘1’ will be given if the criteria are all met. The coder should write a comment if non-dairy milk is featured on the menu as it is expected this will be a rarity. |
|  | iii. Yoghurts should be low sugar | Where branded products are listed, these can be looked up online to check whether they meet the sugar requirements. Those meeting the requirements will receive a ‘1.’ If yoghurts are mentioned but do not state which brands they are or whether they are natural or low in sugar, a ‘0.5’ rating will be given as it is assumed that these are still a good source of calcium – but the coder should make a comment on this. Where there is no mention of any yoghurt, an ‘A’ will be given. A search should be done for ‘yogurt’ or ‘yoghurt’ to ensure that this item is identified. |
|  | iv. No hot drinks (tea, coffee or hot chocolate) | Primary school menus that mention hot drinks on their menus will be awarded a ‘0.’ Menus without any mention of hot drinks will be awarded an ‘A.’ |
| **Oils and spreads** | i. Except potatoes, a portion of food prepared, battered or breaded, deep-fried or flash fried can be provided up to a maximum of twice a week | Discernment is required to identify what foods are likely to be battered, breaded, deep-fried or flash fried. Menus with three or more of these foods will be rated ‘0.’ Those with two or fewer will be rated ‘1.’ It should be assumed that most Chinese dishes (i.e., noodles and ‘sweet and sour’) are flash-fried. Unless otherwise stated, assume that sausages are baked or grilled rather than fried. Also, coders should assume that chicken goujons and fish fingers are breadcrumbed and cooked in the oven. It must be assumed that battered fish is fried. Non-meat and non-dairy foods are not included in this criterion. |
| **HFSS foods** | i. No confectionery or savoury snacks may be provided except crackers, water biscuits and oatcakes | Menus including confectionery and non-permitted savoury snacks will receive ‘0.’ Those without these menu items cited will receive an ‘A.’ Those with snacks (i.e., oatcakes) meeting criteria will receive a ‘1.’ |
|  | ii. Cakes and biscuits are permitted at lunchtime but must not contain any confectionery | Cakes and biscuits containing or topped with confectionery will be awarded a ‘0.’ Those with compliant-sounding cakes and biscuits will also be awarded ‘0’ as this is a healthiness rating code sheet and cakes or biscuits are not typically ‘healthy.’ No mention of any cakes or biscuits on the menu or price list will receive an ‘A.’ |
|  | iii. No salt should be available to pupils to add to food after the cooking process is complete | No mention of salt on the menu will be rated ‘A.’ Menus that explicitly state they have salt available will be rated ‘0.’ |
|  | iv. The portion of any condiment made available to pupils must not exceed 10ml | Failure to mention condiments on the menu will receive an ‘A.’ Menus stating that 10ml condiments are available will receive ‘1.’ A comment should be written if assumptions are made. If condiments sound like a meal component (i.e., sweet chilli dipping sauce) then it is assumed that they exceed the 10ml portion size so would be rated ‘0.’ |
| **Prices** | i. Cost of the meal of the day | If the price is available, the coder should write it in the notes column. |
|  | ii. Notes | Primary school meals are either universally free or a set price. If the price is available then this could be noted, but it is assumed that pupils can choose whatever is on the menu and it will all cost exactly the same for the parent or caregiver. |
| **General observations** | i. Water must be available free of charge to all pupils | This is likely to be an ‘A’ but menus that feature this will be rated ‘1.’ If water is on a primary school menu, it is assumed that there is no separate cost as meals are a set price. In other words, water is ‘free of charge for all pupils’ if water is explicitly stated on a primary school menu. |
|  | ii. Menu design and detail | This is difficult to quantify, so the coder will write qualitative notes. |
|  | iii. Images, photos and graphics | This is difficult to quantify, so the coder will write qualitative notes. |
|  | iv. Descriptive names of menu items (i.e., traditional versus foreign/unusual) | This is difficult to quantify, so the coder will write qualitative notes. The coder may do some counting, e.g., on 3/5 days foods were ‘foreign/unusual’ versus 2/5 days of ‘traditional’ meals. The coder may also comment on the descriptive language around cooking methods, e.g., ‘oven-baked’ or ‘homemade.’ |
|  | v. Notes | This is difficult to quantify at this stage and will be purely qualitative notes. |
