# Supplementary material for: Developing and using a School Menu Healthiness Assessment Tool to analyse school food provision in Wales
Source: Public Health Nutr. 2025 Jan 10;28(1):e36. doi: 10.1017/S1368980025000047 (PMC11822622; doi:10.1017/S1368980025000047)
Supplement: Gilmour and Fairchild supplementary material 2 — Gilmour and Fairchild supplementary material [file S1368980025000047sup002.docx]

B. Secondary school food provision code book

|  | **Description** | **Coding** |
| --- | --- | --- |
| **Breakfast** | i. Menu inclusive of permitted categories | ‘A’ for the absence of any school breakfast menu or price list. Some schools may have a price list or additional information. It should not be assumed that all these foods are available at breakfast, unless it is explicitly stated – so an ‘A’ should be given. ‘1’ rating for menus featuring only foods in the four categories permitted by the *Regulations* (see Justification / Explanation column), regardless of how many categories are fulfilled. Menus containing a non-permitted food or drink will be rated ‘0.’ If bacon is on the breakfast menu, this breakfast category should be rated ‘0.’ |
| **Break time** | i. Fruit and/or vegetables must be available | An ‘A’ will be given where no information is provided about break time provision. Otherwise, ‘0’ if there is break time provision available to rate and there are no fruit and/or vegetables available and ‘1’ if these are available. If there is fruit on the price list, then it may be assumed that this is available at break time. |
|  | ii. No cakes or biscuits permitted | If no information is provided, an ‘A’ will be given. If cakes and biscuits are on the menu, a ‘0’ will be given. Bagels, pikelets, crumpets and teacakes do not fall under the ‘cakes and biscuits’ break time category. It is assumed that tea cakes are not *Tunnock’s* teacakes (biscuit and marshmallow), instead tea cakes are a sweet bread made with dried fruit and are permitted during break time. If cakes and biscuits are on the price list, it should not necessarily be assumed that these are available at break time. |
|  | iii. No confectionery or savoury snacks may be provided, except crackers, water biscuits and oatcakes | ‘A’ will be given where there is no information provided. If confectionery and/or savoury snacks are on the menu, a ‘0’ will be given. If bacon is on the menu, a ‘0’ will be given for this criterion. Coder’s discernment is required to identify what items on a price list are available at break time; for example, a ‘slice of bacon’ may be a break time item whereas a Yorkshire pudding or chicken and chips on the price list may only be available during lunchtime. |
|  | iv. Notes | This will be purely qualitative notes. The coder should write if there are ‘lunchtime’ options available at break time – e.g., pasta, sandwiches, wraps and baguettes. |
| **Fruit and vegetables** | i. At least two portions of vegetables or salad must be provided daily | Provision is to be calculated per day (i.e., if there are four portions on one day, this will only total ‘two’ portions for that day). If the menu generically states, ‘seasonal vegetables’ then it must be assumed that there is sufficient variety and be rated ‘1.’ Otherwise, an incremental decimal of 0.2 from 0 to 1 should be given (5 days in a week) based on the variety available over the 5-day week. E.g., if only ‘peas’ are on the menu five days a week, the rating would be ‘0.2.’ Those with only two different vegetables across the week would receive a ‘0.4’ rating. Menus with a good variety of vegetables across the week, differing daily would receive a ‘1.’ All menus and/or price lists should feature vegetables, so a ‘0’ will be given if these are not seen. The scoring for this criterion is dependent on the quantity (two or more portions available) as well as variety available across the menu. |
|  | ii. At least two portions of fruit, fruit salad or fruit juice must be provided daily | If there is no mention of fruit on the menu or price list, an ‘A’ should be given. Provision is to be calculated per day (i.e., if there are multiple portions on one day, this will total ‘two’ portions for that day. If the menu generically says that fruit is available, it must be assumed that there is variety and a ‘1’ will be given. Otherwise, an incremental decimal of 0.2 from 0 to 1 is to be given (5 days in a week) based on the variety available over the five-day week. Examples as above. If fruit is tinned, make note of this. |
|  | iii. A fruit-based dessert (containing at least 60g raw fruit) at least twice a week | If a dessert appears heavily fruit-based, i.e., apple pie or fruit tart then it is assumed that the raw fruit quota is met. Menus with no fruit-based desserts will receive a ‘0,’ menus with one fruit-based dessert will receive a ‘0.5’ and those with two or more will receive ‘1.’ If there is no mention of any desserts on the menu, this criterion will be coded ‘A.’ The coder should read the menu carefully and do searches for ‘apple’ and ‘crumble’ etc. to ensure these are not missed. Fruit jelly, Eve’s pudding, carrot cake and Welsh cakes contain enough fruit to be counted as a ‘fruit-based dessert.’ However, cheesecake, a lemon drizzle or orange zest cake do not contain enough raw fruit to count as a ‘fruit-based dessert.’ It should not be assumed that ‘homemade desserts’ are fruit-based and meet the criteria. The coder should write a comment if any assumptions are made. |
|  | iv. Fruit or vegetable juice combined with water may be provided | ‘A’ will be given where there is no information provided or information lacks sufficient detail to rate. Otherwise, ‘1’ if provided. |
|  | v. Smoothies made with fruit or vegetable juice and milk, yoghurt, or non-dairy milks may be provided | ‘A’ will be given where there are no smoothies on the menu or price list. Smoothies made with fruit or vegetable juice plus milk, yoghurt and non-dairy milks will receive a ‘1.’ If no information about smoothie ingredients is given, this absence of information should be recorded with an ‘A’ plus a comment saying that smoothies are available. Smoothies with the addition of other ingredients (other than those listed in the criterion) will provide additional calories and will not be scored positively. Hence, smoothies with other additional ingredients (i.e., nut butters, oats) are not permitted and will receive a ‘0.’ |
|  | vi. Smoothies made with blended fruit or vegetable puree may be provided | ‘A’ will be given where there are no smoothies on the menu. If smoothies made with blended fruit or vegetable puree are available, a ‘1’ will be given. If no information about smoothie ingredients are provided, this absence of information should be recorded with an ‘A’ plus a comment saying that smoothies are available. Smoothies with the addition of other ingredients (other than those listed in the criterion) will provide additional calories and should not be scored positively. |
|  | vii. Fruit or vegetable juice drinks must not exceed 15g added sugar per litre | If branded drinks are listed, the sugar content can be checked accordingly. Those below 15g added sugar per litre will receive a ‘1’ and those higher than 15g will receive a ‘0.’ Where no information is provided regarding the brand or drink contents, an ‘A’ will be given. |
| **Meat, fish and alternatives** | i. A portion of fish at least twice a week | Provision is to be calculated per day (i.e., if there are multiple portions on one day, this will total ‘one’ portion). The BBC *Good Food* website must be used to check menu ingredients. Menus with no mention of fish will receive a ‘0’ and those with one portion of fish in a week will receive a ‘0.5.’ Menus with two or more portions of fish per weekly menu will receive a ‘1.’ Information that is not in the menu cycle should be included in the count. If tuna is available (i.e., in a baguette or jacket potato), the menu should be rated ‘1’ as pupils could eat this twice a week if desired. If fish type is not specified, it should be assumed that the fish is non-oily. |
|  | ii. A portion of oily fish at least twice during any four-week period | Menu cycles with no oily fish at all will receive a ‘0’ and those with one portion will receive a ‘0.5’ and those with at least two portions will receive a ’1.’ Menus with one portion per four weeks will receive a ‘0.5.’ To meet the secondary school menu criteria, oily fish must be available once every two-week cycle or twice per three-week cycle. |
|  | iii. Meat cuts on at least three days a week (not including luncheon meat – i.e., sliced ham) | Meat cuts include: any meat or poultry including joints, cooked sliced meat; bacon or mince (ground) meat. Provision is to be calculated per day (i.e., if there are multiple portions on one day, this will total ‘one’ portion). The BBC *Good Food* website must be used to check menu ingredients. Menus with no meat cuts will receive a ‘0,’ those with one meat cut per week will receive a ‘0.33,’ two days of meat cuts ‘0.66’ and three or more days ‘1.’ Menu items coded as a ‘meat cut’ can include red meat cuts – these will be coded twice. |
|  | iv. Red meat a maximum of three days each week | Provision is to be calculated per day (i.e., if there are multiple portions on one day, this will total ‘one’ portion). For example, if there are four types of red meat dish available on a single day, this would count as ‘one portion’ because pupils would typically only consume one item or meal per day. For rating secondary school menus, red meat provision must be across three different days to receive the top mark of ‘1’. The BBC *Good Food* website must be used to check menu ingredients (e.g., Frikadelle is a red meat item). Menus with no red meat will receive a ‘0,’ those with one portion of red meat will receive a ‘0.33,’ those with two portions will receive ‘0.66’ and menus with three portions will receive a ‘1.’ Menus with four or more portions each week should receive a ’0.’ This criterion is focused on lunchtime, so if there is red meat available daily at break time or breakfast these should not be taken into account for this score. If there are different options given for a roast dinner (i.e., turkey, pork, chicken or gammon), it may be assumed that red meat is offered and this would count as a portion of red meat (one out of a possible three across the week). A comment should be written if this assumption is made. Burgers that do not specify the meat are assumed to be beef (red meat) and are not a ‘processed meat’ or ‘meat product.’ Unless otherwise specified, it should be assumed that burritos, lasagne and meatballs contain red meat. Sausages are a ‘processed meat’ and should not be counted as a ‘red meat’ as their meat content can be fairly low. Pepperoni and salami (i.e., as a pizza topping) are counted as a processed meat, but the quantity is too small to also be counted as a ‘red meat.’ |
|  | v. No more than two 'meat products' or ‘processed meat’ portions each week | Provision is to be calculated per day (i.e., if there are multiple portions on one day, this will total ‘one’ portion). The BBC *Good Food* website must be used to check menu ingredients. Menus featuring meat products on two days or fewer will receive a ‘1’ and those with ‘processed meat’ or ‘meat products’ on three or more days will receive a ‘0.’ Sausages are a ‘processed meat’ and should not be counted as a ‘red meat.’ Ham, gammon and bacon are counted as a ‘red meat’ and a ‘processed meat.’ Chicken goujons are a ‘meat product’ or ‘processed meat,’ unless it is specified that these are chicken breast or ‘homemade’ – in which case they would be classified as a ‘meat cut.’ Pepperoni and salami (i.e., as a pizza topping) are counted as a processed meat, but the quantity is too small to also be counted as a ‘red meat.’ Non-meat, non-dairy options are not a ‘processed meat’ or ‘meat product.’ |
|  | vi. For vegetarians / vegans, a portion of non-dairy protein three or more days a week | Provision is to be calculated per day (i.e., if there are multiple portions on one day, this will total ‘one’ portion). It is important to include the price list non-meat, non-dairy protein sources in the count – the extent to which the price list is used may be commented upon. The BBC *Good Food* website should be used to assess whether a menu item is a source of protein: i.e., the BBC *Good Food* vegetarian lasagne has vegetables rather than pulses or soya protein, so it is assumed that vegetarian lasagnes are not a protein source; a vegetarian curry is assumed to contain protein (likely pulses or beans); quesadillas contain cheese so are not a ‘non-dairy protein source.’ Menus without any non-meat, non-dairy protein will be rated ‘0.’ Those with one will be rated ‘0.33’ and those with two will be rated ‘0.66,’ with prevalence of three or more days being rated ‘1.’ Comments should be made if the non-meat, non-dairy options appear to be largely UPF. If the only suitable option is baked beans and a jacket potato available daily, this menu would be rated ‘0.33’ due to a lack of variety. |
| **Potatoes, bread, rice, pasta and other starchy carbohydrates** | i. A portion of potato or potato product cooked in oil is permitted a maximum of twice a week | Menus surpassing this and featuring three or more portions of potato or potato product cooked in oil will receive a ‘0.’ Menus with two or fewer portions of potato or potato product cooked in oil will receive a ‘1.’ Unless otherwise specified, it will always be assumed that roast dinners contain roast potatoes which are cooked in oil. Diced potatoes, wedges and hasselback potatoes are assumed to be cooked in some oil, unless it is stated these are oven-baked (purchased frozen chips and wedges are likely to be pre-cooked in some oil). If the menu description of potatoes is vague, i.e., ‘potato’ or ‘Cooks choice potatoes’ then it should be assumed that these are not cooked in oil as there are numerous other ways in which potatoes can be cooked. |
|  | ii. One or more wholegrain starchy food each week | Menus with no wholegrain starchy foods will be rated ‘0’ and those with at least one wholegrain starchy food across that week will be rated ‘1.’ If there is no mention of wholegrains or this cannot be deciphered, the rating will be an ‘A.’ 50:50 bread may be counted as a wholegrain starchy food, but a note should be written if this is the case. |
|  | iii. At least one portion of starchy carbohydrates (excluding potatoes) must be provided daily | Menus without any starchy carbohydrate alternative to potatoes will receive a ‘0.’ Those with one or more alternative will receive a ‘1.’ |
|  | iv. On days that potatoes cooked in fat/oils are on the menu, there must be an alternative starchy food not cooked in fat/oil available | It may be difficult to decipher this, depending on how detailed the menu is. Menus meeting this criterion will be awarded a ‘1.’ Those not meeting the criteria will be awarded ‘0 and those with insufficient information will be given an ‘A.’ |
| **Dairy and alternatives** | i. Provided milk must be semi-skimmed or skimmed | If there is no mention of milk, an ‘A’ rating will be given and if semi-skimmed or skimmed milk is specified, a ‘1’ rating will be given. If milk is provided but there is no indication as to whether it is semi-skimmed or skimmed, the rating will be ‘0.5.’ Milk can include milkshakes as it is presumed that there is a high volume of dairy in the drink. Any milk, even if only available one or two times a week should be rated ‘0.5’ or ‘1.’ The coder may write a comment about frequency of provision. |
|  | ii. Soya, rice or oat milk must be unsweetened (<5% added sugars) and contain added calcium | No mention of non-dairy milk alternatives will be rated an ‘A.’ If the milks are provided, but there is no description of sweetening or fortification, a ‘0’ will be given. A ‘1’ will be given if the criteria are all met. The coder should write a comment if non-dairy milk is featured on the menu as it is anticipated that this will be a rarity. |
|  | iii. Tea and coffee must only be made with semi-skimmed or skimmed milk | If there is no mention of tea and coffee, or tea and coffee are mentioned without stating what type of milk, the menu will be rated an ‘A’ but the coder should make a comment regardless. If semi-skimmed and skimmed milk are mentioned, the menu rating for this criterion will be ‘1.’ |
|  | iv. Hot chocolate drinks must be up to 250ml and only made with water, semi-skimmed or skimmed dairy milks or <5% added sugar non-dairy milks | Where there is no mention of hot chocolate on the menu, it will receive an ‘A.’ Menus that state hot chocolate is available but provide no details as to how it is made will receive an ‘A’ plus the coder should write a comment. If hot chocolate is listed and meets the criterion, a ‘1’ will be given. |
|  | v. Flavoured milks and/or yoghurts should be low sugar | Where branded products are listed, these can be looked up to check that they meet the sugar requirements. Those meeting the requirements will receive a ‘1.’ If yoghurts and milks are mentioned but do not state which brands they are or whether they are natural or low sugar, a ‘0.5’ rating will be given as it is assumed that these are still a good source of calcium – but the coder should make a comment on this. Where there is no mention of any yoghurt or milk, an ‘A’ will be given. If possible, a search should be done for ‘yogurt’ or ‘yoghurt’ to ensure that it this item is identified. |
| **Oils and spreads** | i. Except potatoes, a portion of food prepared, battered or breaded, deep-fried or flash fried can be provided up to a maximum of twice a week | Discernment is required to identify what foods are likely to be battered, breaded, deep-fried or flash fried. Menus with three or more of these foods will be rated ‘0.’ Those with two or fewer will be rated ‘1.’ It should be assumed that most Chinese dishes (i.e., noodles and ‘sweet and sour’) are flash-fried. Unless otherwise stated, coders must assume that sausages are baked or grilled rather than fried. Also, it should be assumed that chicken goujons and fish fingers are breadcrumbed and cooked in the oven. It should be assumed that battered fish is fried. Non-meat and non-dairy proteins are not included in this criterion. |
| **HFSS foods** | i. No confectionery or savoury snacks may be provided, except crackers, water biscuits and oatcakes | Menus including confectionery and non-permitted savoury snacks will receive ‘0.’ Those without these menu items will receive an ‘A.’ |
|  | ii. Cakes and biscuits must not contain any confectionery | Cakes and biscuits containing or topped with confectionery will be awarded a ‘0.’ Those with compliant-sounding cakes and biscuits will also be awarded ‘0’ as this is a healthiness rating code sheet and cakes or biscuits are not typically ‘healthy.’ No mention of any cakes or biscuits on the menu or price list will receive an ‘A.’ |
|  | iii. Drinks must not exceed 15g added sugar per litre | If branded drinks are listed, the sugar content can be checked online. Those below 15g added sugar per litre will receive a ‘1’ and those higher than 15g will receive a ‘0.’ Where no information is provided, or there are no drinks to analyse, an ‘A’ will be given. The coder may write a comment about which drinks are available. |
|  | iv. No salt should be available to pupils to add to food after the cooking process is complete | No mention of salt on the menu will be rated ‘A.’ Menus that explicitly state they have salt available will be rated ‘0.’ |
|  | v. The portion of any condiment made available to pupils must not exceed 10ml. | Failure to mention condiments on the menu will receive an ‘A.’ Menus stating that 10ml condiments are available will receive ‘1.’ If the price of a condiment is on the price list (i.e., £0.09, £0.10, £0.11 etc.) then it may be assumed that this is for a portioned sachet. A comment should be written if this assumption is made. If condiments sound like a meal component (i.e., sweet chilli dipping sauce) then it is assumed that they exceed the 10ml portion size so would be rated ‘0.’ |
| **Prices** | i. Cost of the meal of the day | If the price is available, the coder should write it in the qualitative notes column. |
|  | ii. Notes | The coder should make notes about the prices of certain foods and also draw conclusions, if possible. For example, the cost of a main meal versus the FSM allowance, the price of sweet-baked products versus fruit, the cost of components to make a balanced meal. The extent of comments is dependent on whether the menu has a price list. |
| **General observations** | i. Water must be available free of charge to all pupils | This is likely to be an ‘A’ if the information is absent, but menus that state water is available for free will be rated ‘1.’ |
|  | ii. Menu design and detail | This is difficult to quantify, so the coder will write qualitative notes. |
|  | iii. Images, photos and graphics | This is difficult to quantify, so the coder will write qualitative notes. |
|  | iv. Descriptive names of menu items (i.e., traditional versus foreign/unusual) | This is difficult to quantify, so the coder will write qualitative notes. The coder may do some counting, e.g., on 3/5 days foods were ‘foreign/unusual’ versus 2/5 days of ‘traditional’ meals. The coder may also comment on the descriptive language around cooking methods, e.g., ‘oven-baked’ or ‘homemade.’ |
|  | v. Notes | The coder will write qualitative notes on relevant areas that were not covered in the above criteria. |
